# Commentary: Changes in Oral Health Policies and Guidelines During the COVID-19 Pandemic

**DOI:** 10.3389/froh.2021.718538

**Published:** 2021-07-08

**Authors:** Sonica Singhal, Julie Farmer, Carlos Quiñonez

**Affiliations:** ^1^Department of Dental Public Health, Faculty of Dentistry, University of Toronto, Toronto, ON, Canada; ^2^Public Health Ontario, Toronto, ON, Canada

**Keywords:** aerosol generating procedures, fallow period, cross infection (MeSH), heating ventilation air conditioning, COVID-19

We commend Jiang et al. for their cross-national collaboration to assimilate information on changes in oral healthcare policies and guidelines developed in response to COVID-19 [1].

During COVID-19, apart from working in closed environments in close proximity to staff and patients, one of the biggest challenges for oral healthcare workers, including in Canada, is to mitigate infection transmission risk while conducting aerosol generating procedures (AGPs) [2, 3]. Given this, Canadian dental regulatory authorities, professional associations, and professionals themselves have been on a constant watch for any COVID-19 outbreaks in dental offices. As a matter of fact, in Canada, multiple episodes of outbreaks in dental offices have surfaced in the media in the last many months [4–9]; however, it appears that none report outbreaks as a result of provider-to-patient or patient-to-provider transmission. On that note, Table 1 of Jiang et al., based on a news article [6], states that Canada witnessed an outbreak in a dental office due to cross infection from dental treatment, which is an inaccurate description of the facts [1]. The news article though stated that “public health officials in Waterloo Region have declared a COVID-19 outbreak at a dental service after three cases were linked to it,” there was no mention of cross-infection due to dental treatment. Further investigation before stating such an important and sensitive information, which has a potential to be detrimental to public and patient perceptions of the safety of dental care in Canada as well as globally, was warranted. This outbreak happened in the Region of Waterloo in the province of Ontario, Canada's most populated province with the largest number of dentists. Through personal communication with the concerned officials at the time of the dental office outbreak, it was confirmed that the outbreak was related to staff-to-staff transmission. There were no high-risk contacts identified outside of the workplace and no high-risk contacts among the patients. The dental setting contacted all of their patients who had been onsite on the days impacted and notified them of a possible low-risk exposure.

On another note, authors state that “*national requirements for heating, ventilation, and air-conditioning (HVAC) systems in health care facilities are determined by the Canadian Standards Association*” (CSA). Importantly, dental clinics are actually not captured under “health care facilities” in Canada, which means that these national requirements do not apply to dentistry; in fact, the CSA has no regulatory power in this context. Further, only one of the four provincial dental regulatory authorities or associations included in the Jiang et al. paper (British Columbia) [10] refer to CSA standards for establishing HVAC systems. In addition, of the four provinces [10–13], guidelines around observance of fallow period are only provided in Ontario [11], based on the Centers for Disease Control and Prevention's table for airborne contaminant removal times [14], and not the CSA standard, as described by Jiang et al.

Finally, while Jiang et al. acknowledge the existence of dental regulatory authorities in Canada's 13 provincial and territorial jurisdictions, they have focussed their findings on only four of these jurisdictions. Notably, variation in the content of these policies has been noted in Canada, one example being described above [15]. The article by Jiang et al. being a nine countries comparison, understandably could not provide comprehensive information on specific countries; that said, Canadian readers might get further benefitted if a more complete review of inter-jurisdictional guidance is planned in the future.

## Author Contributions

SS identified inaccuracies in the aforementioned manuscript, did facts check with the concerned authorities, and prepared the first draft. SS, JF, and CQ conceived the idea of acknowledging the gaps through this commentary and finalized the manuscript. JF reviewed the relevant literature. CQ and JF reviewed and made edits. All authors contributed to the article and approved the submitted version.

## Conflict of Interest

The authors declare that the research was conducted in the absence of any commercial or financial relationships that could be construed as a potential conflict of interest.
